# A Model-Based Evaluation of Noninvasive Biomarkers to Reflect Histological Nonalcoholic Fatty Liver Disease Scores

**DOI:** 10.1007/s11095-024-03791-2

**Published:** 2024-12-19

**Authors:** Iris K. Minichmayr, Elodie L. Plan, Benjamin Weber, Sebastian Ueckert

**Affiliations:** 1 Present Address: Department of Clinical Pharmacology, Medical University of Vienna, Vienna, Austria; 2 Department of Pharmacy, Uppsala University, Uppsala, Sweden; 3 Translational Medicine and Clinical Pharmacology, Boehringer Ingelheim Pharmaceuticals, Inc., Ridgefield, CT USA; 4 Present Address: Pharmetheus, Uppsala, Sweden; 5 Present Address: Global Translation, Novo Nordisk A/S, Måløv, Denmark; 6 Present Address: Ribocure, Mölndal, Sweden

**Keywords:** histological liver scores, item response theory, liver biopsy, model, nonalcoholic fatty liver disease

## Abstract

**Background:**

Nonalcoholic fatty liver disease (NAFLD) comprises multiple heterogeneous pathophysiological conditions commonly evaluated by suboptimal liver biopsies. This study aimed to elucidate the role of 13 diverse histological liver scores in assessing NAFLD disease activity using an *in silico* pharmacometric model-based approach. We further sought to investigate various noninvasive patient characteristics for their ability to reflect all 13 histological scores and the NAFLD activity score (NAS).

**Methods:**

A histological liver score model was built upon 13 biopsy-based pathological features (binary and categorical scores) from the extensive NASH-CRN (Nonalcoholic Steatohepatitis-Clinical Research Network) observational NAFLD Database study (n = 914 adults) using the concept of item response theory. The impact of 69 noninvasive biomarkers potentially reflecting NAFLD activity was quantitatively described across the entire spectrum of all 13 histological scores.

**Results:**

The model suggested that four different disease facets underlie the cardinal NAFLD features (steatosis, inflammation, hepatocellular ballooning (= NAS); fibrosis; highest correlations: corr_ballooning-fibrosis_ = 0.69/corr_inflammation-ballooning_ = 0.62/corr_steatosis-inflammation_ = 0.60). The 13 histological liver scores were best described by contrasting noninvasive biomarkers: Age and platelets best reflected the fibrosis score, while alanine and aspartate aminotransferase best described the NAS, with diverging contributions of the three individual NAS components to the results of the overall NAS.

**Conclusions:**

An *in silico* histological liver score model allowed to simultaneously quantitatively analyze 13 features beyond NAS and fibrosis, characterizing different disease facets underlying NAFLD and revealing the contrasting ability of 69 noninvasive biomarkers to reflect the diverse histological (sub-)scores.

**Graphical Abstract:**

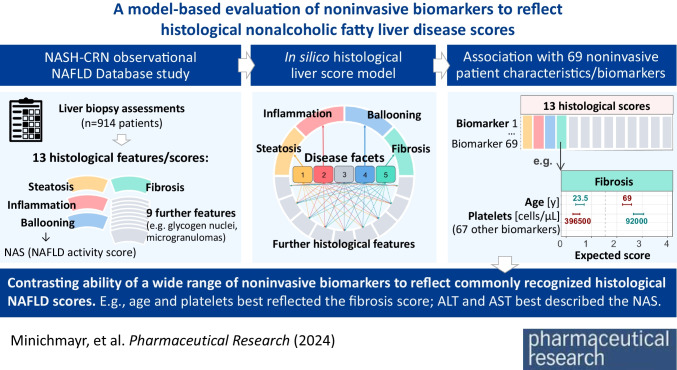

**Supplementary Information:**

The online version contains supplementary material available at 10.1007/s11095-024-03791-2.

## Introduction

Nonalcoholic fatty liver disease (NAFLD), including its progressive subtype nonalcoholic steatohepatitis (NASH), has attained the proportions of a global non‐communicable metabolic pandemic, not least fueled by the staggering parallel rise in obesity and metabolic syndrome worldwide [1]. The increasing clinical and economic burden of NAFLD is aggravated by the lack of approved drug treatments for this disease and has prompted intensive research and numerous studies in a concerted effort to address the unmet medical need of NAFLD. The development of novel therapies, however, has been challenged by the complex ‘multi-hit’ pathophysiology of NAFLD, inconsistent diagnostic criteria, and a lack of clarity about appropriate treatment endpoints [2, 3].

To date, NAFLD and NASH diagnoses and trial endpoints have heavily relied on liver biopsies. NAFLD is defined as hepatic fatty infiltration (i.e. steatosis) of at least 5% in biopsy specimens, but encompasses an entire spectrum of disorders associated with diverse lesions and highly variable pathological manifestations, ranging from simple steatosis (nonalcoholic fatty liver: NAFL) to steatohepatitis (NASH) and cirrhosis [1, 4]. In addition to steatosis, NASH is histologically characterized *inter alia* by the presence of hepatocellular ballooning as well as lobular inflammation and may also involve liver fibrosis [4]. Due to the wide variation also within the diagnosis of NASH and the dynamic nature of NAFLD, experts in the field have recommended to move away from categorizing NAFLD patients into two groups (NASH/non-NASH) and to instead view NAFLD and individual histopathologic features in the context of a spectrum [5].

To evaluate the severity of NAFLD and different single pathological features, a histological scoring system has been developed by the NASH Clinical Research Network (NASH-CRN) [6]. The scoring system comprises 14 histological features, each graded (e.g. steatosis: 0–3) or recorded as present or absent. The sum of the scores for steatosis (0–3), lobular inflammation (0–3) and hepatocellular ballooning (0–2) is referred to as NAFLD activity score (NAS, 0–8) and constitutes a widely, often isolatedly assessed, histological key metric of NAFLD [6]. The (draft) guidance for industry on NASH with liver fibrosis by the U.S. Food and Drug Administration includes (i) the NAS score as a histological efficacy endpoint, and (ii) the fibrosis stage (score 0–4), which to date is considered the major prognostic determinant of clinical outcome of NAFLD [7]. A multitude of clinical trials have assessed one or more histological features of the NASH-CRN scoring system to judge NAFLD severity and/or therapeutic success or to compare patient groups at different ends of one score [8–10]. For example, the histological scoring system served as the foundation of evaluating liver biopsies in the multi-center observational NAFLD Database study, which forms part of the publicly available NIDDK (National Institute of Diabetes and Digestive and Kidney Diseases) NAFLD Adult Database [10].

Although liver biopsies and derived activity scores like NAS are still considered the gold standard for diagnosing and assessing NAFLD and NASH–also in clinical trials–, this approach suffers from various limitations including its invasiveness, associated potential morbidity and life-threatening complications, poor patient acceptance, limited repeatability, technical hurdles including sampling variability and/or error, assessor variability and costs. These drawbacks consequently entail implications for clinical trials, e.g. biopsy-based screening and inclusion criteria or treatment endpoints [11]. To facilitate the assessment of NAFLD and consequently clinical trials, and to at the same time reduce harm for patients, noninvasive biomarkers are urgently required and efforts for their identification are strongly encouraged by regulatory authorities.

Diverse previous studies have investigated potential relationships between biopsy-based features and noninvasive clinical variables. These analyses generally focused on single histological scores (e.g. fibrosis) or even one specific part of a score (e.g. advanced fibrosis, score 2–4 [12]), and on one or few selected patient characteristics (e.g. diabetes [13]). Furthermore, most commonly, traditional statistical tests or group-wise comparisons between specific patient groups have been performed to show the presence/absence of a significant association between noninvasive and histological markers (e.g. a difference in fibrosis between patients with different age or sex), thus providing limited quantitative information [8, 10]. To analyze multiple clinical scores simultaneously, modelling techniques have proven useful, although their application to NAFLD data has been scarce and again limited to single histological scores and their progression over time [14].

Item response theory (IRT) is a statistical methodology–originally developed in the field of psychometrics to analyze educational assessments–that has increasingly been applied to clinical scores quantifying the intensity of different facets of a disease [15]. The fundamental basis of IRT are mathematical models that relate the probability of the outcome of each item in an assessment (e.g. a histological liver score) to one or more so-called latent variables, which capture particular disease aspects (e.g. underlying NAFLD) manifesting in the observed questionnaire-based scores. The strength of IRT-based analyses lies in the models’ inherent ability to combine and implicitly weigh the various discrete observations to yield enriched latent variables on a more informative interval scale. The number of latent variables needed to represent the data relates to the number of constructs, i.e. disease facets, measured by the clinical score-based assessments. The value of applying IRT to healthcare-related measures has been increasingly recognized, as witnessed by an ever-increasing number of therapeutic fields (e.g. Parkinson’s disease, schizophrenia, multiple sclerosis) [15].

The current study aimed to leverage the benefits of IRT to enhance the understanding of the disease aspects underlying NAFLD by quantitatively assessing the role of diverse individual and composite scores of the NASH-CRN liver scoring system–including the NAS score and its components–in determining NAFLD disease activity. In this setting, an item represents a histological liver biopsy feature that will be related to latent variables, with each one capturing a particular aspect underlying NAFLD. Furthermore, we sought to analyze the correlation of these latent variables with a large set of noninvasive biomarkers using a model-based approach, with the goal to identify the noninvasive patient characteristics best reflecting the full spectrum of the NAS, fibrosis and further histological scores.

## Methods

### Study Population and Data

The study population originated from the public NIDDK NAFLD Adult database, more specifically, from the observational NAFLD Database study, which was undertaken by the NASH-CRN and enrolled patients with histologically proven or suspected NAFLD at nine U.S. medical centers between 2004 and 2009 [10, 16]. Data from the Nonalcoholic Fatty Liver Disease (NAFLD) Adult Database reported here are available for request at the NIDDK Central Repository (NIDDK-CR) website, Resources for Research (R4R, https://repository.niddk.nih.gov). All patients enrolled in the NASH-CRN provided written informed consent before data collection. The institutional review board of each participating clinical center and a data safety monitoring board approved the study protocol, consent statements and data collection forms.

Data of 914 adult patients were considered, including biopsy information (1 sample/ individual) as well as an abundance of (n = 69) diverse, scientifically plausible noninvasive patient characteristics, covering (among others) demographic information (e.g. sex), routine clinical and laboratory variables or body size measures (Table I). The raw data containing the relevant histological and biomarker information had been recorded using separate forms (e.g. laboratory results form or physical examination form), often on different days. In case more than one biopsy was available for an individual, the specimen with the smallest time difference to the closest measurement of the liver function tests (‘liver panel’) was considered. For all other biomarkers, the nearest measurement to the selected biopsy was chosen or handled as missing in case the temporal distance between biopsy and biomarker exceeded one year. Individuals without biopsy information were disregarded.

**Table I:** Noninvasive Biomarkers and Clinical Characteristics of the Investigated Population

Biomarker/clinical characteristic	Unit	Median^a^ /Proportion^b^	P_2.5_-P_97.5_^a^	Influential on NAS and/or fibrosis score^c^
General information				n.i
**Age**	**y**	**51.3**	**23.5–69.0**	**FIB**
Sex	% male	35.8	n.a	n.i
Ethnicity	% Hispanic/latino	10.1	n.a	n.i
Family history of NAFLD, NASH^4^	% yes	8.00	n.a	n.i
Family history of cirrhosis^d^	% yes	9.36	n.a	n.i
Family history of diabetes^d^	% yes	57.6	n.a	n.i
Smoking history	% never smoked	49.7	n.a	n.i
Regular smoker^e^	% yes	79.5	n.a	n.i
**Diabetes**^**f**^	**% yes**	**38.1**	**n.a**	**FIB**
Ascites^f^	% yes	1.31	n.a	n.i
**Portal hypertension**^**f**^	**% yes**	**3.61**	**n.a**	**FIB**
Polycystic ovary syndrome^f^	% yes	6.57	n.a	n.i
Antihyperlipidemic medication^7^	% yes	37.0	n.a	n.i
**Cardiovascular or antihypertensive medication**^**g**^	**% yes**	**53.4**	**n.a**	**FIB**
Laboratory results				
**Bilirubin (direct)**	**mg/dL**	**0.1**	**0–0.4**	**FIB**
Bilirubin (total)	mg/dL	0.7	0.2–2	n.i
**Alanine aminotransferase (ALT)**	**U/L**	**55.5**	**16–214**	**NAS**
**Aspartate aminotransferase (AST)**	**U/L**	**40.5**	**16–144**	**NAS, FIB**
**AST/ALT**		**0.77**	**0.39–1.65**	**FIB**
**Alkaline phosphatase**	**U/L**	**82**	**44–187**	**FIB**
**Gamma glutamyl transferase (GGT)**	**U/L**	**49**	**15–317**	**FIB**
Total protein	g/dL	7.2	6.1–8.5	n.i
Albumin	g/dL	4.2	3.4–5	n.i
Prothrombin time	sec	11.7	8.9–14.9	n.i
**International normalised ratio (INR)**		**1**	**0.9–1.3**	**FIB**
**α-fetoprotein**	**ng/mL**	**3.6**	**1.3–13.2**	**FIB**
Triglycerides	mg/dL	151	52.3–456	n.i
**Total cholesterol**	**mg/dL**	**190**	**119–286**	**FIB**
HDL cholesterol	mg/dL	42	25–73.8	n.i
LDL cholesterol	mg/dL	116	50–195	n.i
Serum glucose	mg/dL	98	70.2–235	n.i
**Serum insulin**	**µU/mL**	**17.6**	**4.17–74.1**	**FIB**
**HbA1c**	**%**	**5.8**	**4.7–10.1**	**FIB**
**Homeostatic model assessment for insulin resistance (HOMA-IR)**		**4.36**	**1.02–23.2**	**FIB**
Metabolic syndrome^h^	% yes	73.4	n.a	n.i
**Hemoglobin**	**g/dL**	**14.2**	**11.2–17.3**	**FIB**
**Hematocrit**	**%**	**42**	**34.3–50**	**FIB**
White blood cells (WBC)	10^9^ cells/L	6.6	3.8–11.9	n.i
**Platelet count**	**cells/µL**	**239000**	**92000–396500**	**FIB**
Sodium	mEq/L	140	135–145	n.i
Potassium	mEq/L	4.1	3.4–4.9	n.i
Chloride	mEq/L	103	97–109	n.i
Bicarbonate	mEq/L	27	21–31	n.i
Calcium	mg/dL	9.4	8.6–10.3	n.i
Phosphate	mg/dL	3.6	2.5–4.7	n.i
Blood urea nitrogen (BUN)	mg/dL	13	6–24	n.i
Creatinine	mg/dL	0.8	0.5–1.3	n.i
Uric acid	mg/dL	5.8	3.4–9	n.i
Iron	μg/dL	86	35–168	n.i
Ferritin	ng/mL	140	16–898	n.i
Total iron binding capacity	μg/dL	370	247–520	n.i
**α-1 antitrypsin**	**mg/dL**	**143**	**81.9–214**	**FIB**
Antinuclear antibody (ANA)	% positive	24.1	n.a	n.i
Antismooth muscle antibody (ASMA)	% positive	15.8	n.a	n.i
Antimitochondrial antibody (AMA)	% positive	1.33	n.a	n.i
Thyroid stimulating hormone	μU/mL	1.75	0.28–5.86	n.i
Physical examination				
Weight	kg	95.0	63–139	n.i
Height	cm	166	151–188	n.i
BMI	kg/m^2^	33.7	24.8–48.5	n.i
Waist circumference	cm	108	85.3–135	n.i
Hip circumference	cm	115	95.0–146	n.i
Waist/hip ratio		0.94	0.79–1.09	n.i
Mid-upper arm circumference	cm	35.6	27.1–46.9	n.i
Skin fold	mm	33	10.6–69.8	n.i
Blood pressure, systolic	mmHg	131	105–163	n.i
Blood pressure, diastolic	mmHg	76	56–96.7	n.i
Resting radial pulse	beats/min	74	53–102	n.i
Respiratory rate	breaths/min	17	14–24	n.i
Temperature	centigrade	36.6	35.7–37.3	n.i

^a^for continuous covariates; ^b^for categorical covariates; ^c^covariates with 2.5^th^-97.5^th^ range covering ≥ 25% of the expected score (NAFLD activity score NAS or fibrosis score FIB; marked in bold); n.i. not influential according to criterion stated in ^c^; n.a. not applicable; percentages refer to total respondents (patients with missing values or answers not considered); ^d^Any of the patient’s first-degree relatives (parent, brother, sister, child) has fatty liver disease (NAFLD, NASH) / cirrhosis / diabetes (type 1 or type 2; percentage is based on patients who definitely knew); ^e^Regular smoking of cigarettes defined as smoking ≥ 20 packs of cigarettes in a lifetime or ≥ 1 cigarette a day for one year; ^f^Diabetes: patient has been diagnosed with and treated for diabetes 1 or 2 (diabetes 1: 0.55%) / ascites / portal hypertension / polycystic ovary syndrome previously. ^g^Patient has taken antihyperlipidemic medications / cardiovascular or antihypertensive medications in the past six months. ^h^Metabolic syndrome was defined according to IDF (International Diabetes Federation) criteria

### Histological Liver Score Model

To quantitatively describe the spectrum of NAFLD severity, a histological liver score model following the item response theory paradigm was developed based on biopsy assessments, which had been conducted by the NASH-CRN Pathology Committee according to the NASH-CRN histology scoring system. The scoring system comprised five histological features with ordered score categories (steatosis and lobular inflammation: 0–3; hepatocellular ballooning and portal chronic inflammation: 0–2; fibrosis: 0–4), eight features recorded as absent or present (0 or 1: microvesicular steatosis, microgranulomas, large lipogranulomas, acidophil bodies, pigmented macrophages, megamitochondria, Mallory's hyaline, glycogen nuclei) and one qualitative feature describing the location of a lesion [6]. For analysis, levels 1a, 1b and 1c of the fibrosis score were summarized (score = 1).

All 13 ordered and binary histological subscores, reflecting and quantifying the grade of different pathological lesions of NAFLD as manifestations of heterogeneous underlying disease aspects, were jointly integrated in an item response theory model with multiple latent variables. For each histological criterion of liver assessment (i.e. item), an item model mapped the subject-specific latent variable values to response probabilities in accordance with the nature of the outcome (two-parameter logit models for binary items and graded-response models for ordered categorical items [15]). Histological liver score models assuming one *versus* more underlying disease aspects were investigated, together with potential correlations, which represent a core feature of a multidimensional item response theory models. In addition, the results from an exploratory analysis, freely estimating latent variable-item associations, served as a guide to determine the structure of the final model. Model parameters reflected different qualities of the histological subscores, including their sensitivity to changes of the disease (i.e. their ability to differentiate between low and high disease intensity) and in a wider sense their informativeness for the assessment of NAFLD severity. For further details on the concepts and assumptions of item response theory models, the reader is referred to pertinent expert information [15].

Modelling activities were conducted using R3.6.1 and the R package *mirt* (multidimensional item response theory) using the MHRM (Metropolis–Hastings Robbins-Monro) estimation method [17]. Model evaluation and discrimination were performed by numerical and visual diagnostics, including simulation-based assessments (e.g. *n* = 500 simulations to compare observed and simulated correlations between item responses, mirror plots).

### Assessment of Noninvasive Biomarkers

To identify the noninvasive clinical biomarkers and other patient characteristics best reflecting the biopsy-based disease scores (e.g. NAS) and thus NAFLD activity, correlations between the latent variables and the biomarker observations were estimated using a model-based approach. This full random effects modelling approach has previously proven useful in situations with plentiful, potentially correlated covariates and/or missing values [18]. Such models simultaneously capture the relationships among latent variables (i.e., between the different aspects of NAFLD), among multiple noninvasive biomarkers, and, most importantly, between the latent variables and the noninvasive biomarkers. In brief, the distribution of the biomarkers was modelled jointly as random effects and the impact on all latent variables was quantified by estimating a correlation matrix. For example, a high impact of a biomarker on a given latent variable translates to a high absolute value of the corresponding correlation. Furthermore, the correlation is positive when a high biomarker value is associated with a high disease activity and negative for an inverse relationship. A non-parametric bootstrap was used to obtain the imprecision of the estimated correlations (*n* = 200 re-sampled datasets).

The expected histological scores (e.g. NAS and fibrosis) were calculated conditional on the lower and upper ‘end’ of the continuous covariates (i.e. 2.5^th^-97.5^th^ percentile) or for different groups of categorical covariates. Ideally, the expected histological scores given these covariate ‘extremes’ would span the full range of the score (e.g. 0–8 rather than 3–5 in case of the NAS score). The impact of relevant, predictive biomarkers was not merely investigated for the composite NAS score, but also singly for the subscores of its elements (steatosis, inflammation, ballooning), the fibrosis score, as well as all nine residual histological characteristics (items).

The estimation of the correlation between latent variables and biomarkers as well as the non-parametric bootstrap were performed using NONMEM7.4 (ICON Clinical Research LLC, Gaithersburg, MD) assisted by PsN 4.10.0 (https://uupharmacometrics.github.io/PsN). Calculations of associations between histological and noninvasive characteristics and visualization of results were accomplished in R3.6.1.

## Results

### Study Population and Data

**Fig. 1:**
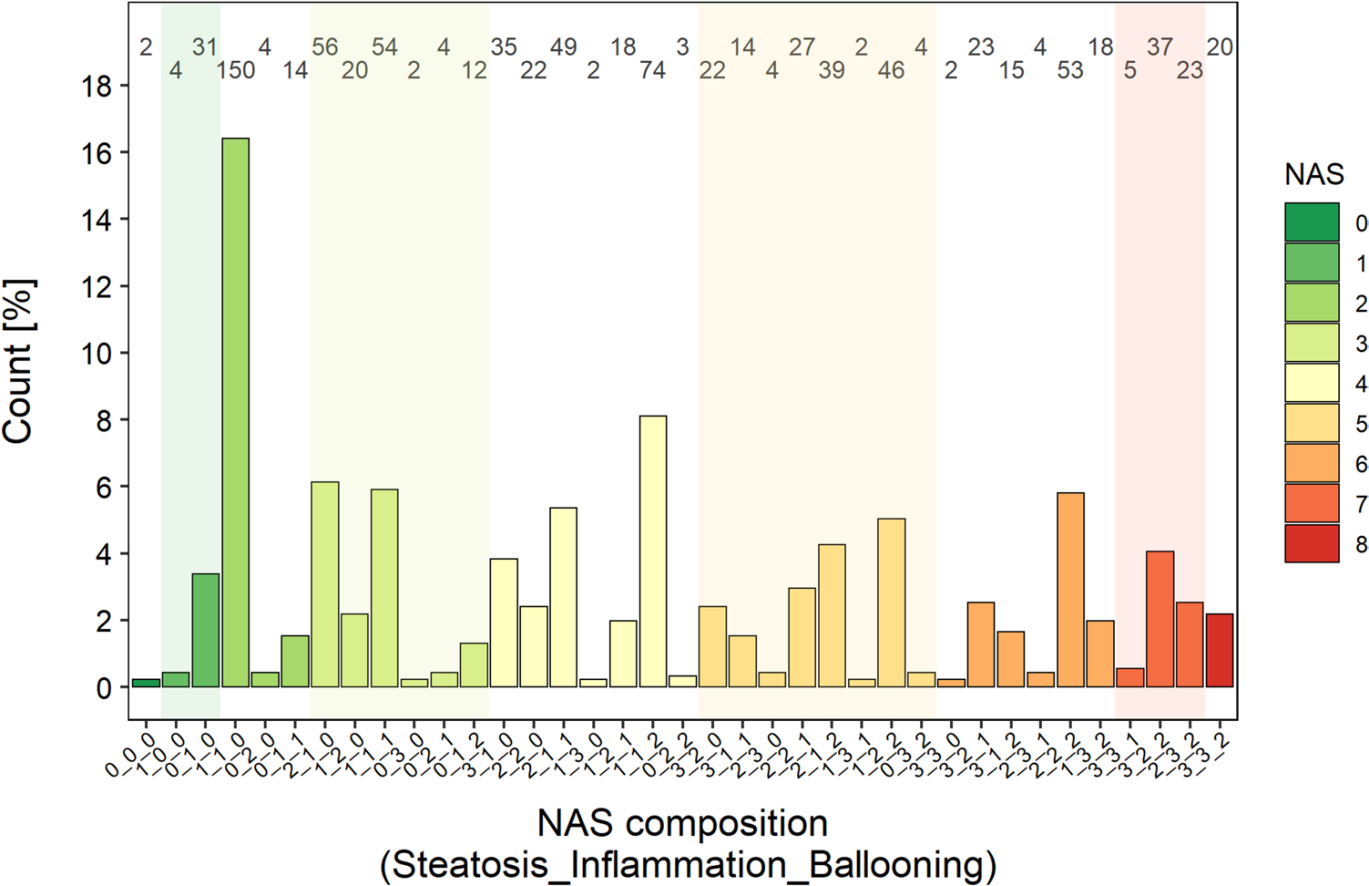
Relative occurrence and composition of the NAS (NAFLD activity score) observed in the study population, ordered by total NAS (increasing values from left to right) and, within one level, by the scores of the NAS elements steatosis – inflammation – ballooning (denoted on the x axis as steatosis_inflammation_ballooning). Colors of the bars and shaded/white areas mark different total NAS scores. The numbers at the top end of the plot represent the absolute count of specific NAS compositions (shown on the x axis) in the study population.

The study population of 914 individuals spanned the full spectrum of liver fibrosis (0–4) and NAFLD (NAS 0–8) (Fig. 1 and S1, Online Resource). Furthermore, almost all possible compositions (i.e. proportions of steatosis/inflammation/ballooning) resulting in the same specific NAS value were covered (Fig. 1). Frequencies of all grades of the 13 histological liver scores are depicted in Fig. S1 (Online Resource). More than half (52.3%) of the patients had obtained a definite positive and 26.8% a definite negative diagnosis of NASH. Patients were as diverse with respect to the measured noninvasive characteristics and biomarkers as regarding their clinical NAFLD manifestation (Table I). For example, patients displayed an age of 28.6–67.0 years (P_0.05_-P_0.95_; median: 51.3 years), a total body weight of 68.1–131 kg (median: 95.0 kg), aspartate aminotransferase (AST) values between 19.0–121 U/l (median: 40.5 U/l), and alanine aminotransferase (ALT) between 9–174 U/l (median: 55.5 U/l). The proportion of missing values of the investigated covariates was generally low (0–34.4%), except for α-fetoprotein (56.9%).

### Histological Liver Score Model

**Fig. 2:**
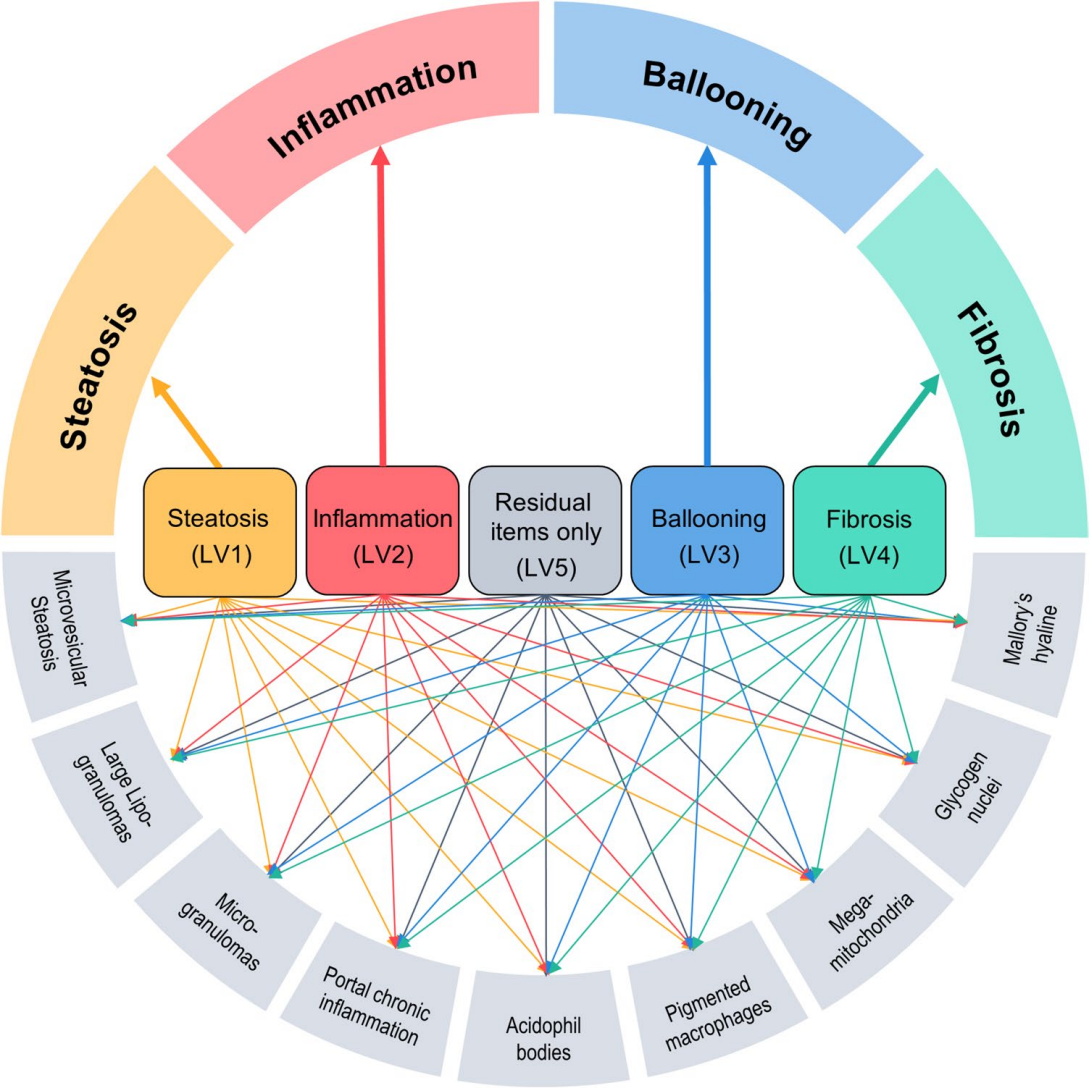
Structure of the histological liver score model. Elements in the circle represent the 13 items (histological liver scores) in the model. LV: Latent variable.

**Fig. 3:**
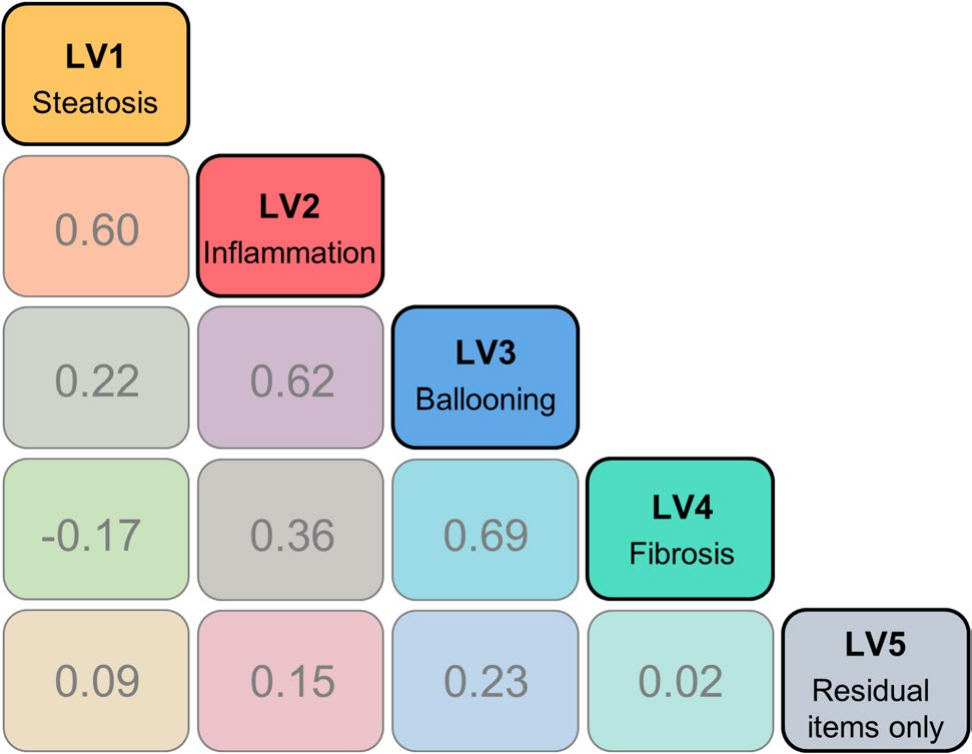
Correlations between the latent disease aspects (represented by numbers in the figure). LV: Latent variable; color codes carry qualitative rather than quantitative information.

The histological liver score model (Fig. 2; Table SI in the Online Resource) suggested five underlying disease aspects of NAFLD to have provoked the documented grades of the 13 histological features of interest. In more technical words, the model best described the data when accommodating five separate latent variables, i.e. four separate latent variables exclusively linked to each of the four cardinal features of NAFLD (steatosis, inflammation, ballooning and fibrosis), and additionally to the nine other histological features, and one latent variable merely covering the residual histological features (but none of the four cardinal features). For the latter features, the model involving the least assumptions performed best, i.e. allowing associations of the residual histological features with each latent variable. Assuming merely one disease construct underlying the histological scores (i.e. one single latent variable) was not even sufficient to adequately describe the three components of the NAS score. Highest correlation on the latent variable scale was identified between the disease aspects reflected by hepatocellular ballooning and fibrosis (69%; Fig. 3).

The model enabled to quantitatively assess the NAFLD disease status of each patient as well as the role of the diverse individual histological metrics in assessing NAFLD severity. Of the five ordered scores, hepatocellular ballooning best differentiated between low and high disease intensity and appeared most sensitive to changes of the underlying disease aspect. In other words, changes in the disease activity related to ballooning led to larger changes of the ballooning score compared to the other features and their underlying disease facet. Patients diagnosed with NASH expectedly tended to have higher disease activity.

**Fig. 4:**
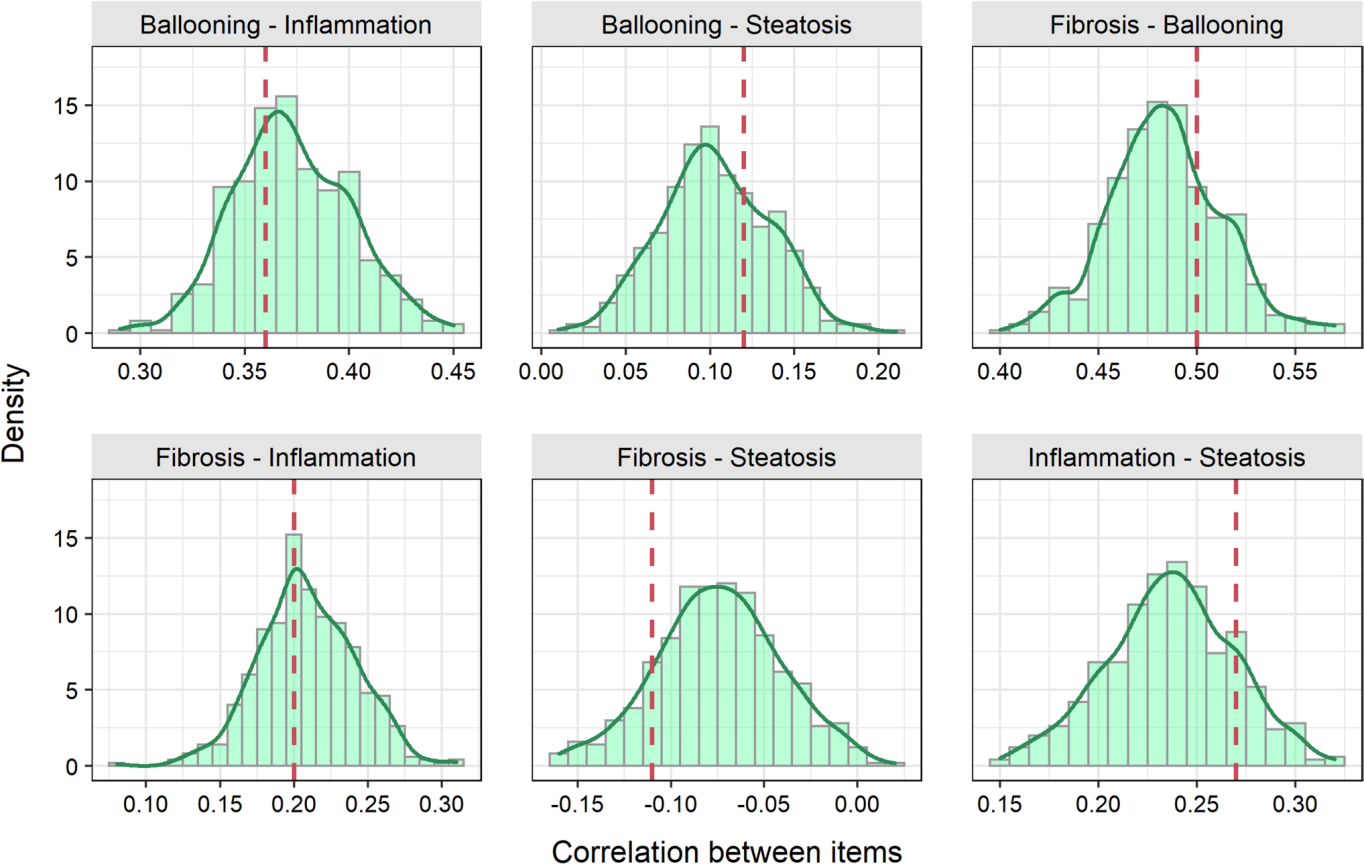
Correlations between histological liver scores (components of the NAFLD activity score NAS and fibrosis) based on 500 simulated (green histograms) and observed (red line) data. Results for all histological liver scores are presented in Fig. S2 (Online Resource). Close values of correlation in the observed data and the mode of correlations in the simulated data indicate good predictive performance.

Model evaluation indicated adequate performance of the developed multidimensional item response model by mimicking different metrics of the observed data (Fig. 4, Fig. S2 and S3 in the Online Resource). For instance, correlations between item responses observed in the raw data could be replicated by stochastic simulations with the model, indicating good predictive performance (Fig. 4 and Fig. S2 in the Online Resource).

### Assessment of Noninvasive Biomarkers

**Fig. 5:**
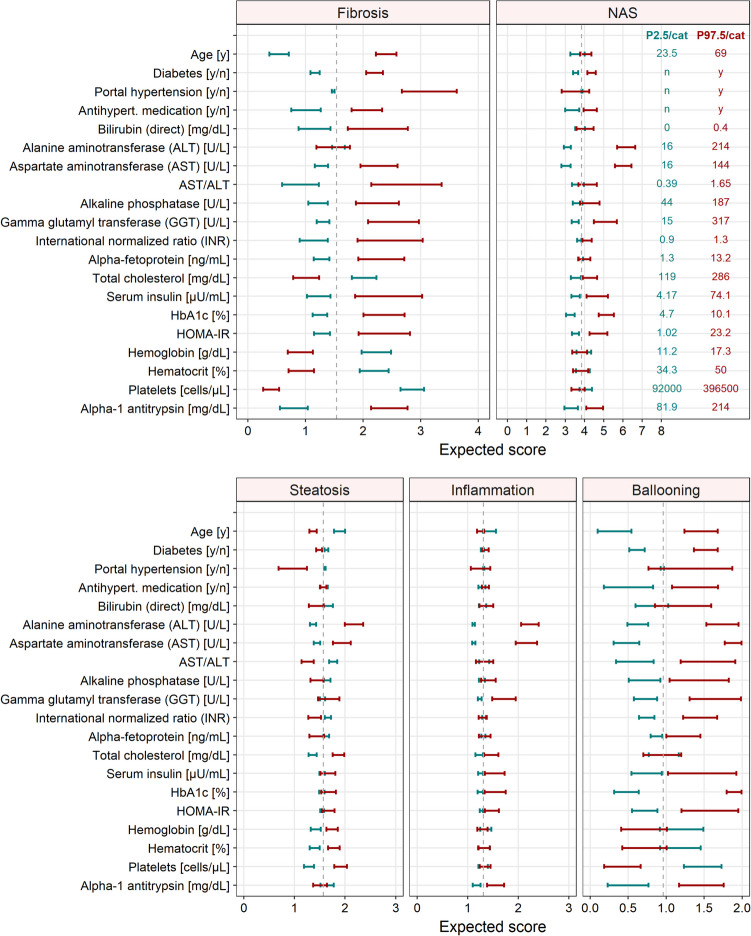
Impact of selected noninvasive biomarkers on the expected NAFLD activity score (NAS) and fibrosis score (upper panel) and on the components of the NAS score (lower panel). Green/red bars represent expected scores and associated uncertainty given the 2.5^th^/97.5^th^ percentile of the covariate or categories (cat) of the covariate (values are stated in the upper right panel); vertical dashed lines depict mean scores. The plots present the biomarkers best predicting the NAS or fibrosis (sub-)scores, i.e. with their 2.5^th^-97.5^th^ percentile covering at least 25% of the expected score range. Results for the same selection of noninvasive biomarkers and the residual nine histological liver scores are depicted in Fig. S4 (Online Resource). The impact of all investigated noninvasive biomarkers and patient characteristics on the NAS and fibrosis score are presented in Fig. S5 (Online Resource).

Distinct noninvasive biomarkers were markedly correlated with different biopsy features quantifying NAFLD severity. The biomarkers that best reflected the NAS or fibrosis (sub-)scores, i.e. with their 2.5^th^-97.5^th^ percentile (Table I) covering at least 25% of the mean expected score range, are depicted in Fig. 5 and described below. Fig. S4 (Online Resource) indicates associations between these selected biomarkers and the nine other histological feature scores studied.

Age and platelets constituted the best descriptors of fibrosis. The analysis suggested a higher expected fibrosis stage, i.e. more advanced disease, in patients displaying higher age and–due to an inverse relationship–lower platelet concentrations. For example, a subject with a platelet concentration of 396,500/μL (97.5^th^ percentile of the platelet distribution), would display an expected fibrosis score of 0.27. The AST/ALT ratio reflected the fibrosis score considerably better than the single biomarkers AST and ALT alone, the latter of which was not predictive of fibrosis.

ALT and AST best described the NAS score, followed by hemoglobin A1c (HbA1c). These findings, illustrating the association of biomarkers with the NAS score, in fact represent a summary of the–partly diverging–results for each NAS component. Whereas AST and ALT–as the best predictors of the overall NAS score–exerted a marked influence on all three single NAS components, results were more heterogeneous for other biomarkers. For the inflammation score, for example, only liver enzymes (AST, ALT, gamma glutamyl transferase GGT) appeared as important descriptors, while steatosis was further associated with platelets, portal hypertension, or cholesterol, though to a lesser extent. In contrast, HbA1c, diabetes and insulin resistance (HOMA-IR: homeostatic model assessment for insulin resistance) appeared highly predictive only of hepatocellular ballooning, which consequently propagated to the NAS score.

As for the nine remaining investigated features of the histological scoring system (Fig. S4 in the Online Resource), significant relationships were found *inter alia* between (i) acidophil bodies and AST as well as ALT, (ii) large lipogranulomas and age as well as platelets, (iii) Mallory's hyaline and e.g. AST, ALT, GGT, α1-antitrypsin as well as HbA1c, glucose and platelets, (iv) portal chronic inflammation and platelets, portal hypertension, as well as (with < 25% coverage of the score range) age and AST/ALT, and also between (v) microgranulomas and portal hypertension. For (vi-xi) glycogen nuclei, megamitochondria, microvesicular steatosis and pigmented macrophages, none of the investigated biomarkers exerted a plausible and marked influence. A summary illustrating the impact of all 69 noninvasive patient characteristics on the NAS and fibrosis score is presented in Fig. S5 (Online Resource). The investigated biomarkers generally covered a broader span of the fibrosis score (overall range: 0.3–3.6 of 0–4) compared to the NAS score (2.2–6.6 of 0–8), with a tendency to mirror high scores better than low scores. Of the three NAS components, the hepatocellular ballooning score was found to be most sensitive to changes of the biomarkers and its range was most widely covered (Fig. 5).

Of all 13 investigated histological features, biomarkers related to diabetes and insulin resistance were predominantly indicative of fibrosis and ballooning (diabetes, HbA1c, glucose, insulin, HOMA-IR), of Mallory's hyaline (HbA1c, glucose) and, to a lower extent, of the NAS score (HbA1c). As for lipids, total cholesterol was associated with fibrosis and, though to a minor extent, steatosis and portal chronic inflammation, while triglycerides were related to ballooning. LDL and HDL cholesterol appeared less informative. Ferritin as an indicator of iron overload appeared modestly associated with ballooning as well as acidophil bodies and inflammation. Sex did not appear to reflect the severity of fibrosis or NAS (see Fig. S5 in the Online Resource).

## Discussion

The developed histological liver score model and subsequent biomarker analysis conveyed valuable insights into NAFLD as well as biopsy-based and noninvasive features of disease evaluation from a hitherto unexplored perspective.

### Histological Liver Score Model

Utilizing the concepts of item response theory, 13 clinical liver scores quantifying the intensity of histologically disparate NAFLD lesions were jointly analyzed to assess the disease qualities underlying NAFLD in a quantitative manner. Our analysis suggested several different disease facets (i.e. separate latent variables) to provoke the observed values of the histological scoring system, reflecting the complexity and multifactorial nature of the disease, i.e., with lesions emerging from different pathophysiological mechanisms. The model structure with four cardinal features (items) reflecting one disease facet each, acknowledging the heterogeneous injury pattern, corresponded well with the NASH-CRN grouping of histological features into five categories (steatosis, inflammation, hepatocellular injury, fibrosis, and miscellaneous features) [6].

The histological liver score model allowed to determine each patient’s disease state and enhances the understanding of the role of the diverse individual histological metrics in assessing disease severity. Such quantitative assessments are challenging particularly given the resource-intensive and limited nature of biopsy data. Thus, the well-characterized ‘real-world’ patient cohort of the NASH-CRN Adult NAFLD database constituted a valuable basis for our analysis, given the variety and myriad of available histological and noninvasive patient features and grades that, importantly, all stemmed from the same patients. Moreover, the quality of the biopsy assessments can be valued high owing to their central review by an expert committee [10].

### Assessment of Noninvasive Biomarkers

A primary focus of the analysis was the identification of noninvasive biomarkers predicting the biopsy-based scores. While previous studies explored potential relationships between clinical variables and NAFLD, even based on NASH-CRN data, they focused on different, commonly smaller subsets. Furthermore, they generally examined one single histological score (e.g. NAS, fibrosis stage or portal chronic inflammation [9, 12, 19]), even one specific grade or part of one score (e.g. only advanced fibrosis/cirrhosis, i.e. score 2–4 [12]), and/or were limited to one or few highly selected patient characteristics (e.g. age/elderly, ethnicity, or aminotransferase levels [8, 12, 20]). Most commonly, they reported the presence or absence of statistical associations between histological and patient features or conducted group-wise comparisons (e.g. NASH/non-NASH) [10]. In contrast, the present analysis addressed one of the declared key objectives of NASH-CRN, i.e. finding associations between clinical and histological features, from a different angle, applying a rather young concept to the field of NAFLD. The combination of item response with full random effects modelling enabled to consider multiple histological scores (13 rather than one), the ranges of a broad palette of clinical characteristics and biomarkers, as well as their influence on the entire observed spectrum of each histological score (e.g. fibrosis score 0–4). This approach acknowledges that NAFLD assessment rests on a collection of multiple histological features rather than any single feature [19] and also aligns with the growing recognition of NAFLD as a spectrum of histopathological lesions [5].

This analysis illustrates the impact of 69 clinical characteristics (Table I) on less commonly addressed histological features beyond NAS and fibrosis. A stringent pre-selection of biomarkers, as commonly used to identify correlated characteristics and identify the most informative ones, was not necessary, as potential correlations between the markers do not pose a problem with the modelling approach taken. The influence of these biomarkers on the histological scores differed between the 13 lesions investigated.

For liver fibrosis, a central factor in NAFLD prognosis, our study identified thrombocytes and age as the best predictors, consistent with previous evidence for advanced fibrosis [8, 21]. A NASH-CRN key publication demonstrated associations of demographic, clinical and laboratory variables with advanced (stage 3 or 4) fibrosis for a different subset of patients, e.g. including those from the treatment trial PIVENS [10]. While several findings aligned with our results, comparability is limited owing to distinct data analysis approaches (associations with specific score grades *versus* full scores), patients and clinical and histological variables. Both studies indicated associations between fibrosis and age, platelets, diabetes, hypertension, waist circumference, hematocrit and, to a less pronounced extent in the current study, prothrombin time, albumin and white blood cells. The main difference concerned ALT, which–unlike AST–did not significantly predict fibrosis in our study. Previous studies also supported the superiority of AST over ALT in detecting (advanced) fibrosis in NAFLD patients [22] and the limited value of ALT in predicting fibrosis [23], possibly due to frequently normal ALT values within the reference range even in patients with advanced NAFLD, or declining ALT with age [12, 24, 25]. Noteworthy, the AST/ALT ratio better reflected the fibrosis score than AST or ALT alone, consistent with its use in several risk scores for advanced fibrosis [26].

The NASH-CRN key publication also reported indicators of definite NASH, suggesting a significant association with ballooning [10]. In our study, most clinical variables reflecting ballooning also appeared as significant NASH predictors in the previous study, except for age and international normalized ratio (INR). These deviations might stem from NASH being diagnosed based on multiple characteristics, although ballooning plays a key role.

The similar impact of the most relevant noninvasive biomarkers on ballooning and fibrosis reflects the 69% correlation of the disease activities associated with these features in the model. This strong interrelationship supports the idea that hepatocellular injury triggers fibrogenesis and fibrosis progression [27]. Fibrosis was also correlated with portal inflammation, which–like fibrosis and ballooning–was also better described by platelets and age [28], fitting its role as a marker of histologically advanced disease, similar to fibrosis [9, 29].

As the second key feature in NAFLD assessment apart from fibrosis, the NAS score, largely covering features of active injury, was best reflected by AST and ALT, unlike fibrosis. Among its subcomponents, inflammation was found to be associated with fewest biomarkers (only liver enzymes), and ballooning with most biomarkers. The diversity of results appears plausible since the NAS score combines three very diverse features, i.e. inflammation and ballooning as indicators of disease activity, and steatosis, which is regarded as an early-stage marker of NAFLD but is considered a less reliable indicator of disease severity [30]. Hepatocellular ballooning resulted most sensitive to varied biomarker values and changes in the underlying disease activity manifesting in ballooning. Our analysis thus supports previous investigations that emphasized the value of ballooning in assessing NAFLD severity, and expressed concern about its lower weight in the NAS score compared to steatosis and inflammation [31].

NAFLD is closely tied to metabolic and cardiovascular disorders such as diabetes type 2, dyslipidemia, obesity or hypertension. For a more detailed discussion of these conditions and the impact of diabetes, body size descriptors, cholesterol, α1-antitrypsin and hypertension on histological liver scores, as well as contextualization of the results within previous literature, the interested reader is referred to the Supplementary Material.

Diagnostic noninvasive biomarkers well representing the status or grade of NAFLD (e.g. advanced disease) without the need for a liver biopsy could enable more frequent and firm diagnoses and consequently a better grasp of the general NAFLD prevalence, but also refine and improve study endpoints and patient selection to better match eligibility criteria. While further investigations are necessary before single or combinations of noninvasive biomarkers can satisfactorily replace biopsies, the current findings offer useful hints on which patients likely display NAFLD and should thus undergo biopsy or innovative diagnostic procedures.

Some limitations have to be acknowledged regarding the present investigation. First, the analyzed demographic and noninvasive biomarkers, albeit abundant, were naturally limited to those available in the NASH-CRN Adult NAFLD database. While the large majority represented routinely measured, readily available, fairly inexpensive and clinically relevant variables, innovative biomarkers have emerged from newer technologies, e.g. imaging techniques like elastography, markers of cellular injury and apoptosis (e.g. cytokeratine-18), or fibrogenesis (e.g. hyaluronic acid or PRO-C3) [1, 32, 33]. Although these present interesting avenues for future studies, their scarce availability and implementation would still limit their use.

Second, some histological (particularly binary) features were imbalanced in the population, with one group dominating. The finding that biomarkers tended to better cover higher score values (particularly for the NAS score and its components), is partly attributable to their higher prevalence (e.g., only 0.7% of the population displayed grade 0 inflammation). This calls to mind that the results are valid for the current study population and data situation–especially given the few representatives for certain scores (e.g., absent inflammation)–and warrants caution against generalizing or transferring them to other populations. Given the importance of identifying patients with advanced disease for treatment and clinical studies, the lower coverage of absent or mild symptoms seems acceptable. As shown in Fig. 5, the lower and upper bounds of the histological scores were generally not captured by the investigated noninvasive biomarkers (unless the majority of the population displayed an ‘extreme’ grade), which might arise from the distribution of score grades, the depiction of the 2.5^th^-97.5^th^ percentiles of the biomarkers (rather than extreme values), uncertainty in the data, or variations in histological staging, even if carried out by experts. The heterogeneous occurrence of scores may have affected the ability of noninvasive biomarkers to demonstrate their impact at both population extremes of the scores. However, the natural history database nevertheless represents clinically plausible real-world scenarios.

Next, an analysis of longitudinal disease patterns, i.e. changes over time, was not supported due to the nature of the available data, which included merely one biopsy for 82.8% of patients and a maximum of three samples in only 1.42% of patients. Future emerging NAFLD registries (e.g. NASH-CRN Database 2 observational study, European NAFLD Registry [34]) might serve to additionally address disease progression in the model. These novel databases may also be utilized to evaluate or update the current model, e.g. with respect to novel biomarkers, refined histological staging systems (as e.g. discussed for ballooning degeneration), or automated machine-learning-based scoring methods [30, 35]. Furthermore, other probabilistic modelling approaches, or less complex methods like confirmatory factor analysis may be explored for comparison. Last, as the correlations between the different disease facets and their associations with biomarkers might change for different situations, such as drug treatment, multidimensional IRT models may be useful for investigating which aspects of the disease are most influenced by a certain treatment strategy.

## Conclusions

An *in silico* histological liver score model based on item response theory enabled the integrated analysis of the full range of 13 diverse histological features (including the widely used NAS and fibrosis scores), measuring both active injury (e.g. hepatocellular ballooning) and slowly progressing lesions (e.g. fibrosis), and their association with 69 noninvasive biomarkers. Our analysis revealed five distinct disease facets underlying NAFLD and the measured histological scores in patients from the NASH-CRN Adult NAFLD database. The ability of multiple clinically relevant noninvasive biomarkers to reflect NAFLD activity could be quantified across the entire spectrum of all 13 histological scores–rather than focusing on one single histological score or a specific part of it using traditional statistical tests, providing limited quantitative information–and was found to differ for distinct biopsy features, with ballooning being the dominant NAS component. The findings offer insights into which patients likely display NAFLD and might thus require biopsy; however, further research is warranted before noninvasive biomarkers can inform study inclusion criteria and endpoints. NAFLD-IRT models may lay the basis for future investigations on the sensitivity of histological liver scores to detect changes of different disease qualities (e.g. inflammation in response to a therapeutic–e.g. anti-inflammatory or antifibrotic–intervention), and on the value of noninvasive biomarkers for evaluating NAFLD treatment options, ultimately providing a step toward more accessible and informative measures beyond the NAS.

## Supplementary Information

Below is the link to the electronic supplementary material.

Supplementary file1 (PDF 592 KB)

## Data Availability

The analysed data originated from the Nonalcoholic Fatty Liver Disease (NAFLD) Adult Database (NAFLD Adult). Data from the Nonalcoholic Fatty Liver Disease (NAFLD) Adult Database [10.58020/53bk-jk73] reported here are available for request at the NIDDK Central Repository (NIDDK-CR) website, Resources for Research (R4R), (https://repository.niddk.nih.gov/studies/nafld_adult).
