# Supplementary material for: A Model-Based Evaluation of Noninvasive Biomarkers to Reflect Histological Nonalcoholic Fatty Liver Disease Scores
Source: Pharm Res. 2024 Dec 19;42(1):123–35. doi: 10.1007/s11095-024-03791-2 (PMC11785690; doi:10.1007/s11095-024-03791-2)
Supplement: Supplementary file 1 — Supplementary file1 (PDF 592 KB) [file 11095_2024_3791_MOESM1_ESM.pdf]

Supporting Information

Pharmaceutical Research

A model-based evaluation of noninvasive biomarkers to reflect histological nonalcoholic fatty liver disease scores

Iris K. Minichmayr[1,2], Elodie L. Plan[2]*, Benjamin Weber[3], Sebastian Ueckert[2]

[1]Dept. of Clinical Pharmacology, Medical University of Vienna, Vienna, Austria

[2]Department of Pharmacy, Uppsala University, Uppsala, Sweden

[3]Translational Medicine and Clinical Pharmacology, Boehringer Ingelheim Pharmaceuticals, Inc., Ridgefield, Connecticut, USA

*Corresponding author:
Mailing address: Box 580, 75123 Uppsala, Sweden
Tel: +46 18-471 4009; Fax: -
Email: elodie.plan@farmaci.uu.se

Table SI Parameter estimates of the histological liver score model

Histological score ('item')	a1[a]	a2	a3	a4	a5	d1[b]	d2	d3	d4
**Ordered categorical histological scores (>2 levels)**									
**Steatosis**	1.287	0	0	0	0	3.026	-0.051	-1.887	-
**Inflammation**	0	2.707	0	0	0	8.413	-0.863	-4.212	-
**Ballooning**	0	0	6.031	0	0	1.721	-2.057	-	-
**Fibrosis**	0	0	0	3.161	0	2.108	-0.090	-1.686	-4.046
**Portal chronic inflammation**	-0.537	0.668	-0.870	2.104	0.526	3.039	-1.817	-	-
**Binary histological scores (present/absent)**						**d**	-	-	-
**Microvesicular steatosis**	3.839	-2.268	-0.411	3.316	0.435	-5.031	-	-	-
**Microgranulomas**	1.201	7.752	-2.456	-2.463	3.009	5.922	-	-	-
**Large lipogranulomas**	-0.004	0.113	-0.346	0.826	0.698	-0.411	-	-	-
**Acidophil bodies**	-0.180	1.156	0.037	0.051	0.544	-1.264	-	-	-
**Pigmented macrophages**	0.142	3.076	-1.238	-1.098	2.892	4.402	-	-	-
**Megamitochondria**	1.258	-1.063	0.305	1.274	0.297	-2.208	-	-	-
**Mallory's hyaline**	-0.881	1.379	5.754	0.621	-0.403	-4.300	-	-	-
**Glyogen nuclei**	0.098	-0.201	0.350	-0.321	0.029	0.167	-	-	-

[a] *a* parameters represent how specific a histological feature is, i.e. how well it can differentiate between patients with high/low disease activity, and thus exhibits its sensitivity to rates of changes caused by the disease (parameter values are stated as relative to variance; latent variables were assumed to be distributed around mean=0 and variance=1)

[b] *d* parameters quantify the activity of a disease aspect (latent variable) required for categorization into a specific category (e.g. steatosis score=1 or steatosis score=3).

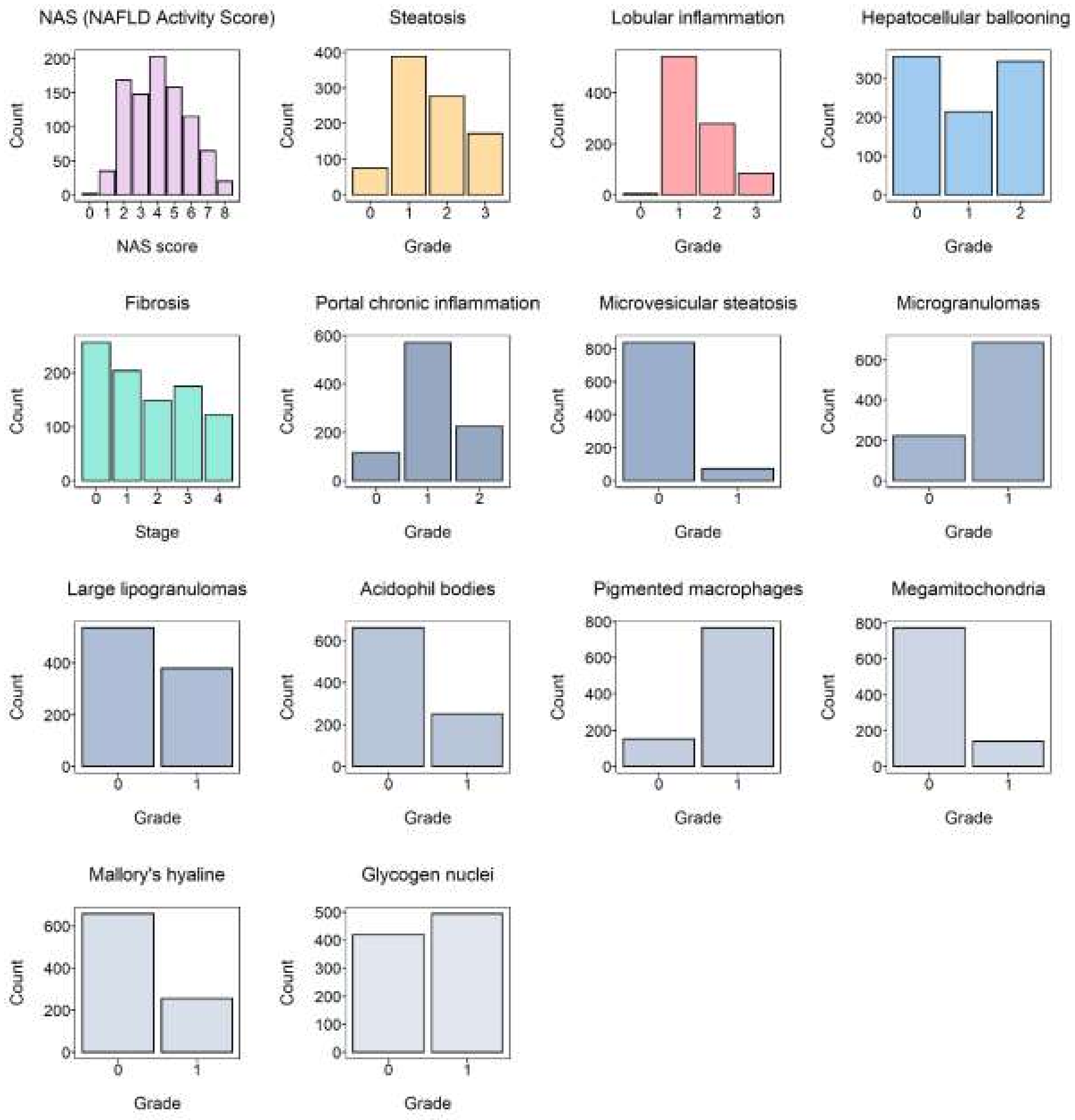

Fig. S1 Distribution of the composite NAFLD activity score (NAS) and 13 individual histological features in the study population (n=914)

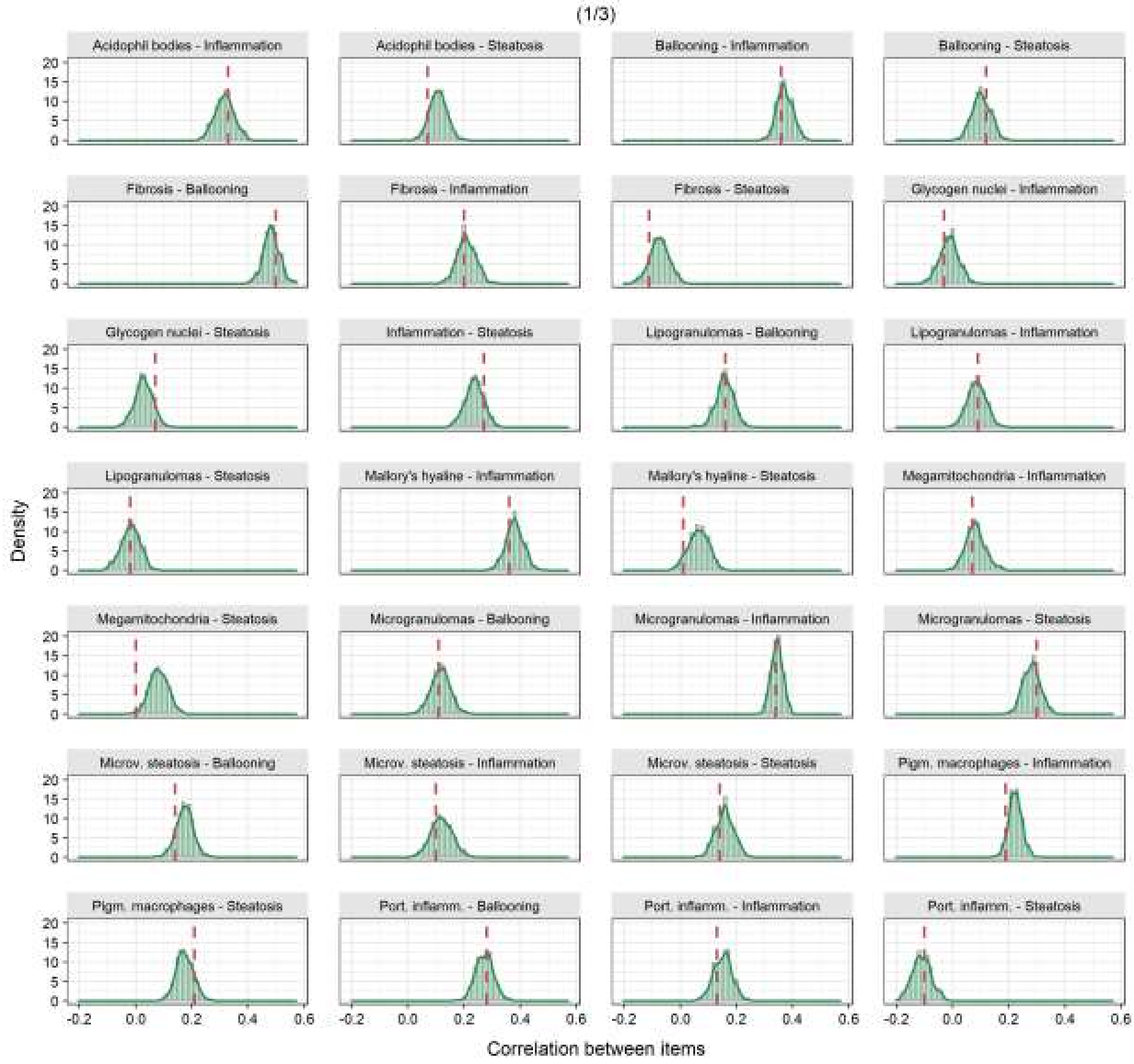

Fig. S2 (1/3) Correlations between histological liver scores based on 500 simulated (green histograms) and observed (red line) data. Items: Steatosis; inflammation; ballooning; fibrosis; microvesicular steatosis; microgranulomas; large lipogranulomas; portal, chronic inflammation; acidophil bodies; pigmented macrophages; megamitochondria; Mallory's hyaline; glycogen nuclei

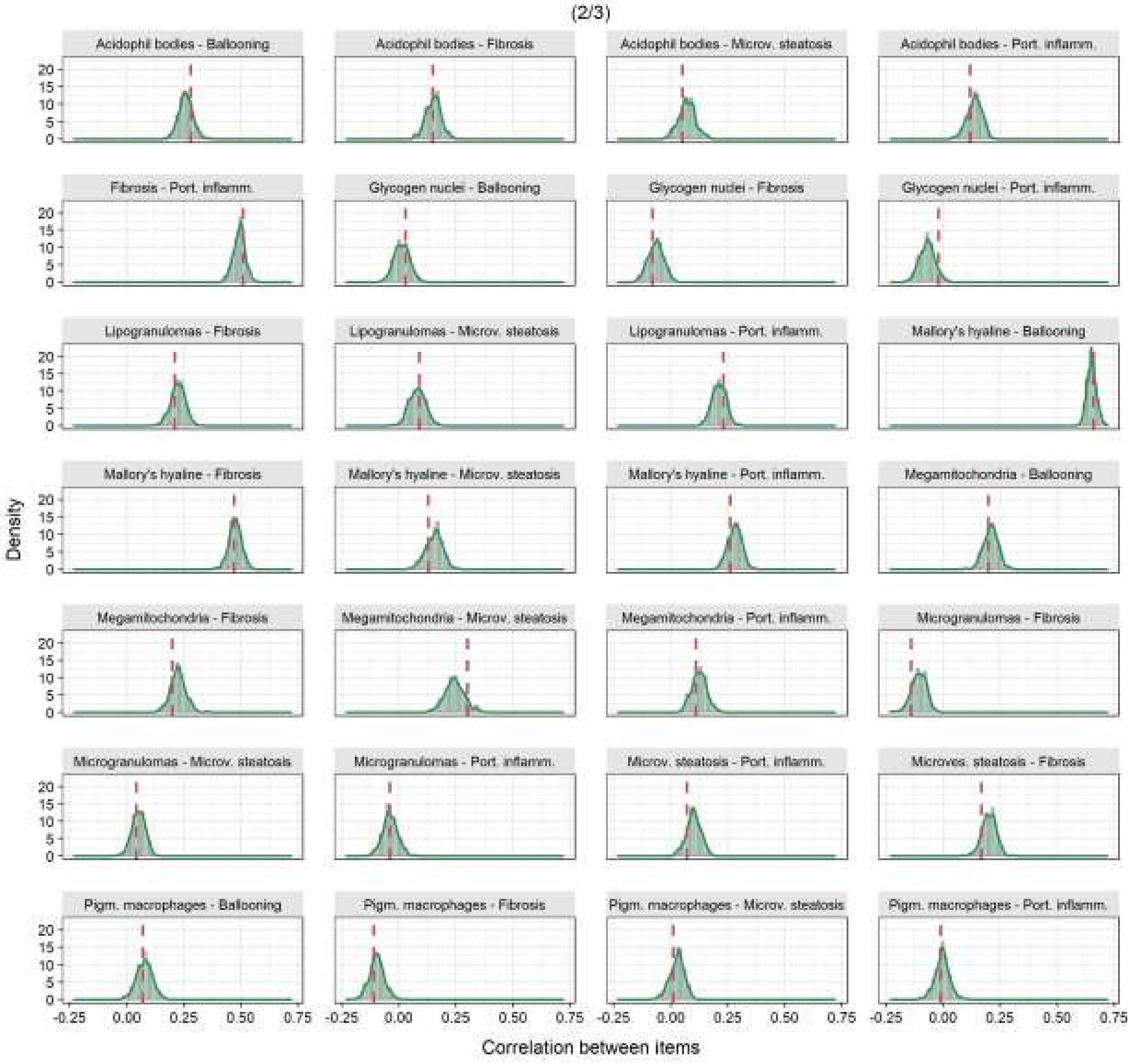

Fig. S2 (2/3) Correlations between histological liver scores based on 500 simulated (green histograms) and observed (red line) data. Items: Steatosis; inflammation; ballooning; fibrosis; microvesicular steatosis; microgranulomas; large lipogranulomas; portal, chronic inflammation; acidophil bodies; pigmented macrophages; megamitochondria; Mallory’s hyaline; glycogen nuclei

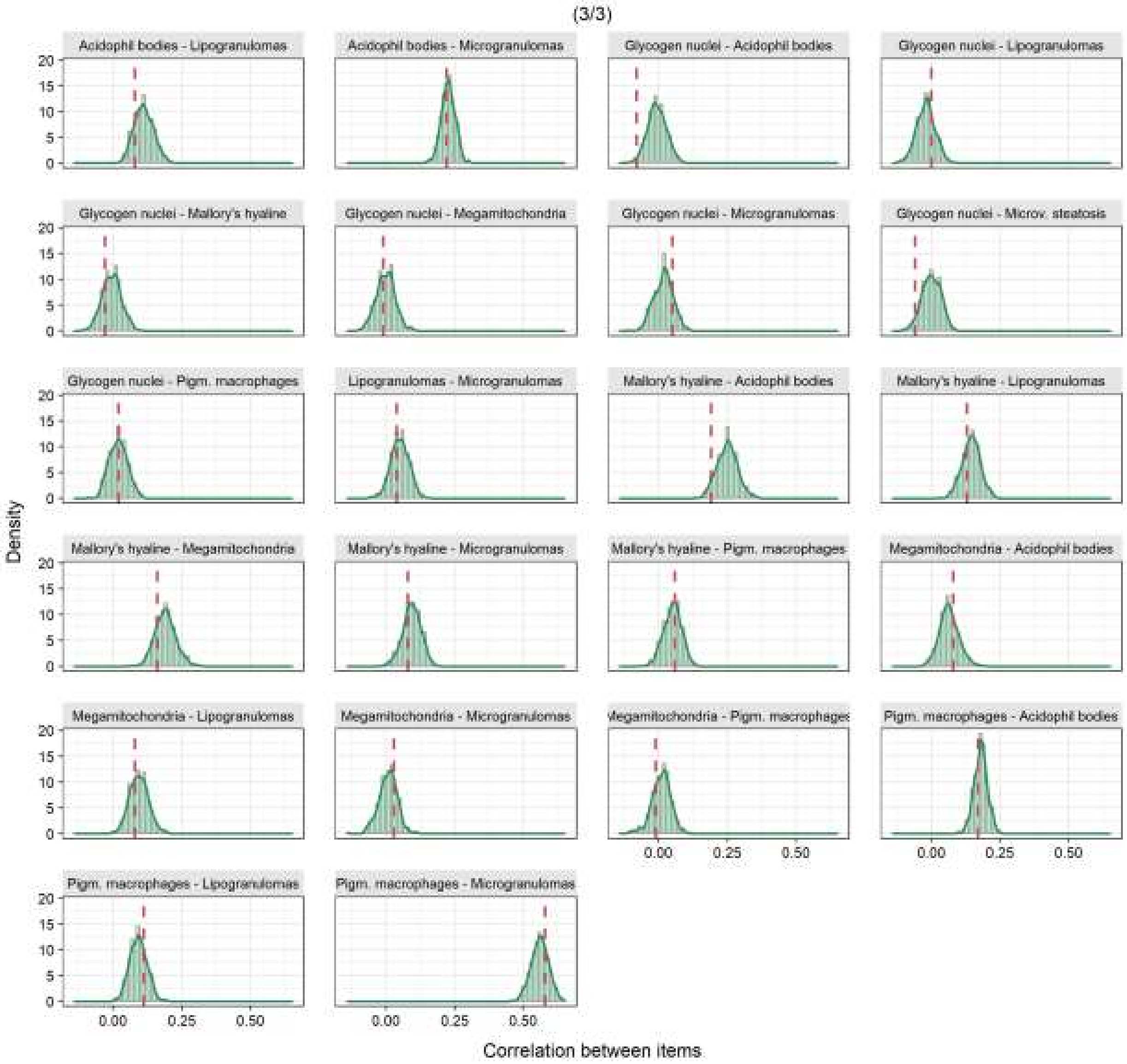

Fig. S2 (3/3) Correlations between histological liver scores based on 500 simulated (green histograms) and observed (red line) data. Items: Steatosis; inflammation; ballooning; fibrosis; microvesicular steatosis; microgranulomas; large lipogranulomas; portal, chronic inflammation; acidophil bodies; pigmented macrophages; megamitochondria; Mallory's hyaline; glycogen nuclei

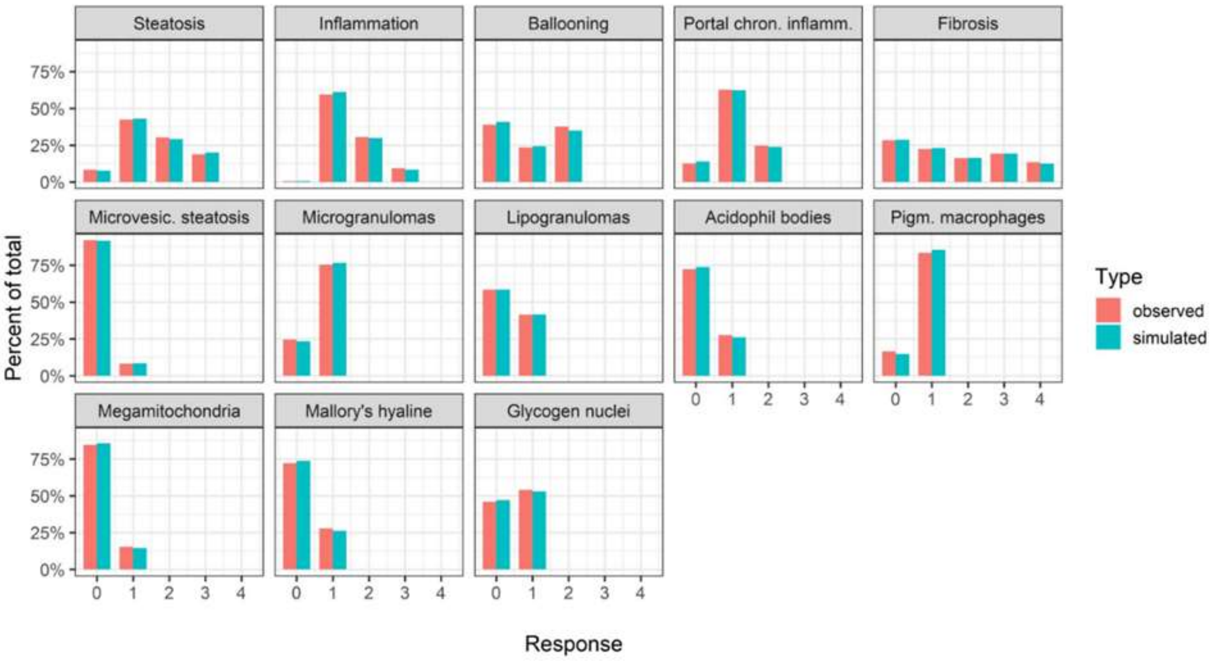

Fig. S3 Mirror plot, goodness-of-fit diagnostic visualizing the distribution of observed responses together with the distribution of a simulation from the model

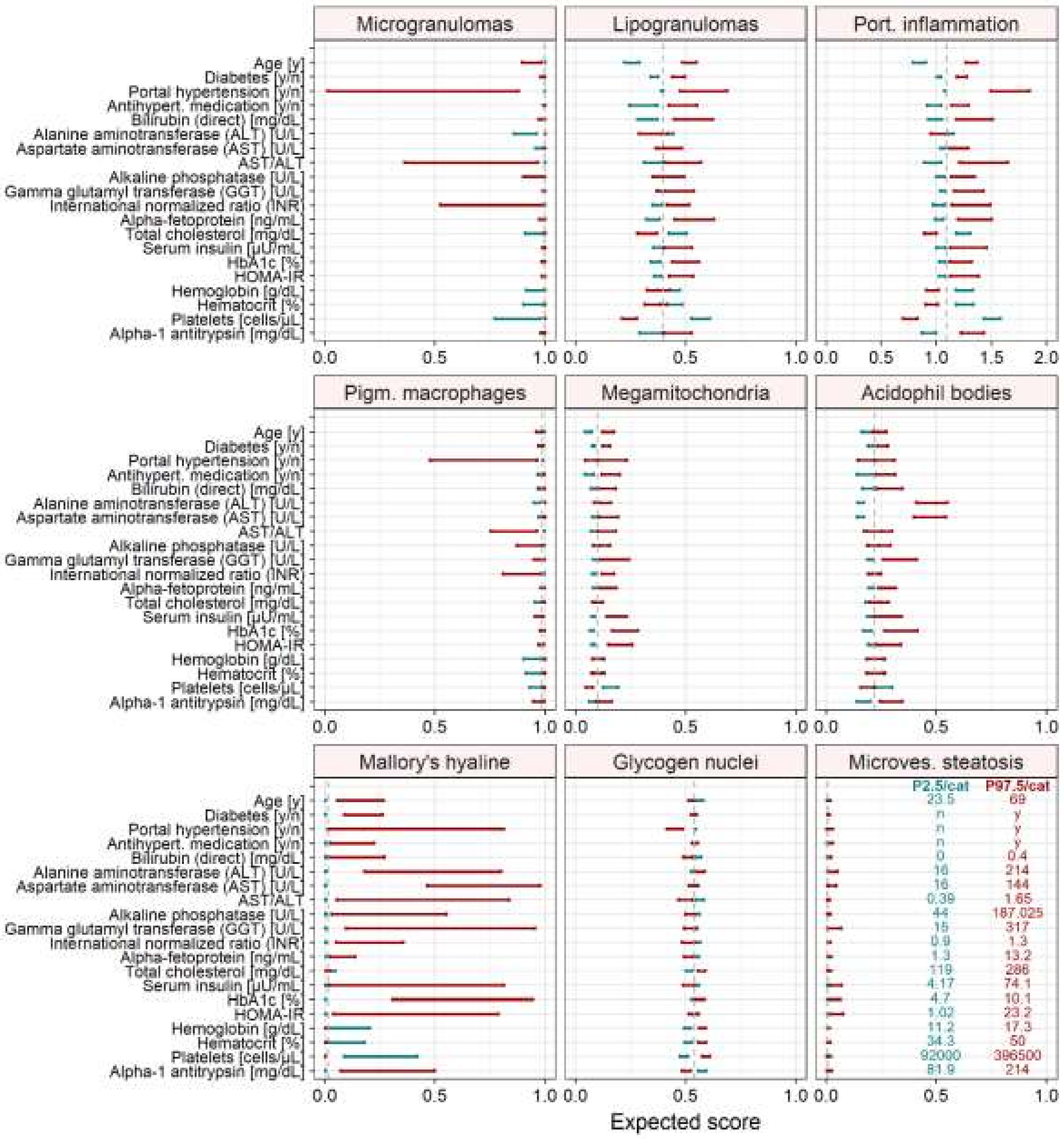

Fig. S4 Impact of selected noninvasive biomarkers on the expected scores of nine histological features (components of the NAFLD activity score and fibrosis are shown in Figure 5). Green/red bars represent expected scores and associated uncertainty given the 2.5th/97.5th percentile of the covariate (values stated in the lower right panel); vertical dashed lines depict mean scores. The plots depict the biomarkers best predicting the NAS or fibrosis (sub-)scores, i.e. with their 2.5th-97.5th percentile covering at least 25% of the expected score range

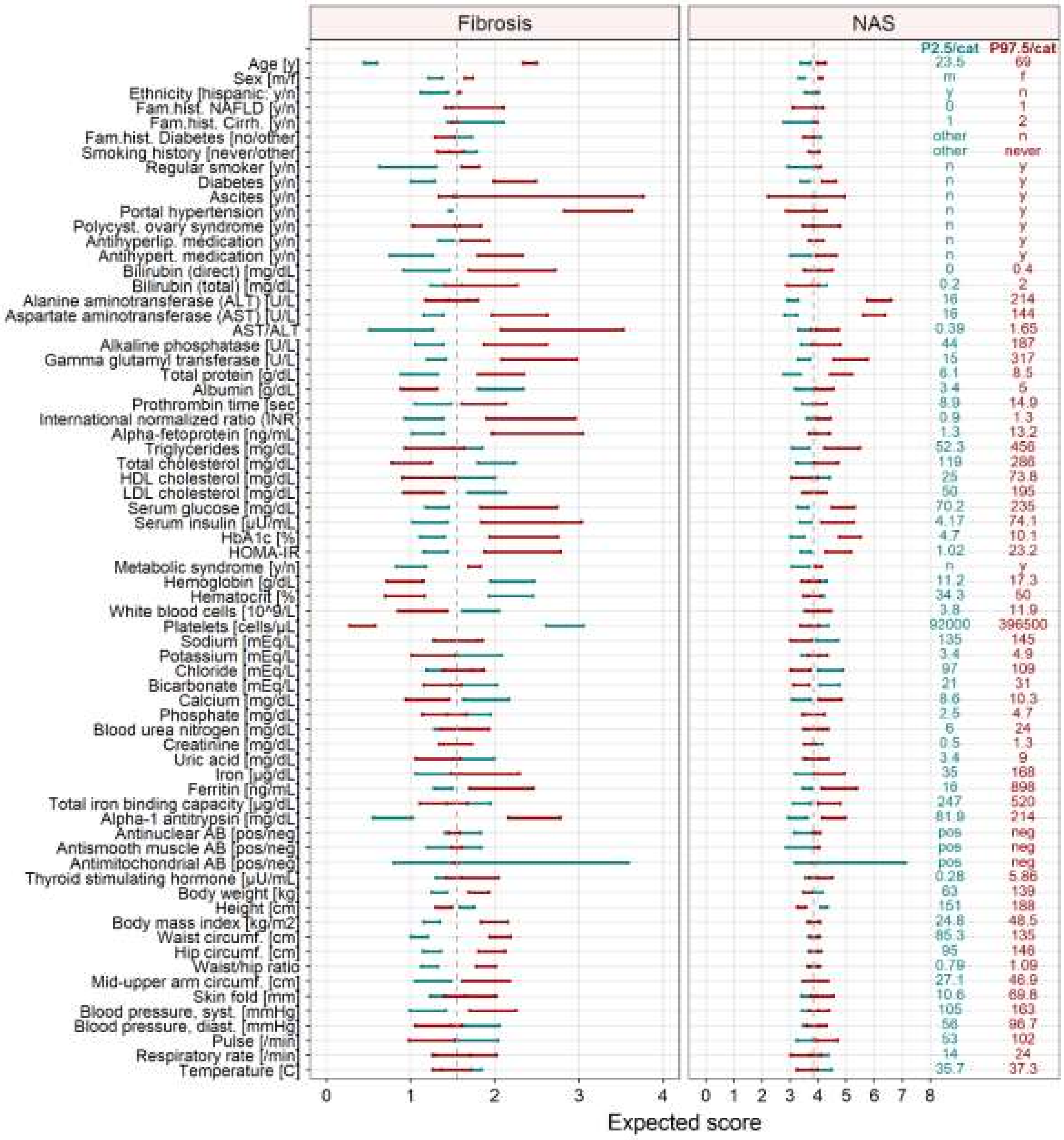

Fig. S5: Impact of the 69 investigated noninvasive biomarkers and patient characteristics on the expected NAFLD activity score (NAS) and fibrosis score. For continuous covariates, the green/red bars illustrate the 95% score uncertainty given the 2.5th and 97.5th percentile of the biomarker. For categorical covariates, the green/red bars represent different groups ('cat'). Vertical dashed lines depict mean scores

Extended discussion on the assessment of noninvasive biomarkers

NAFLD is strongly associated with metabolic and cardiovascular conditions, including type 2 diabetes, dyslipidemia, obesity, and hypertension. Whereas a personal history of **diabetes** has previously been associated with fibrosis (1,2), the current analysis provides a systematic investigation of different factors related to diabetes and glucose metabolism (diabetes, glucose, HbA1c, insulin, HOMA-IR) for their impact on histological scores beyond fibrosis. HbA1c, diabetes and glucose stood out particularly in predicting ballooning, but also fibrosis, Mallory's hyaline and the NAS score (for which HbA1c resulted as the best descriptor after ALT and AST). Although the clinical variable 'diabetes' covered type I and II in the current analysis, virtually identical results were obtained when only focusing on type II diabetes, as these patients constituted the vast majority of the population.

Body size descriptors (e.g. body mass index BMI) as surrogates of obesity overall did not describe the histological liver scores (including steatosis) notably well, with waist circumference-fibrosis appearing as the most relevant relationship. The study population to a large majority consisted of obese patients ($P_{2.5}$=24.8 kg/m^2), impairing the comparison of high and low BMI values regarding their impact on histological features. Of note, although obesity is strongly linked to NAFLD, the disease also affects lean patients and Wang et al. even reported a higher prevalence of ballooning and advanced fibrosis in non-obese patients (3). Besides, metabolic markers like intrahepatic triglycerides have been suggested to better mirror metabolic abnormalities rather than BMI (4).

In agreement with the NASH-CRN key study (5), total **cholesterol** was correlated with fibrosis in the current study; however, superiority of LDL over HDL was not found. Higher total cholesterol was associated with higher steatosis, though–unexpectedly at first view–with lower fibrosis scores. Potential statin therapy in patients with fibrosis might underlie this inverse relationship and, as concomitant drug therapy was not documented in detail in the NASH-CRN database, this finding would need further confirmation. However, a recent study supports our result by highlighting significantly lower total cholesterol in patients with advanced fibrosis despite a similar proportion receiving statin therapy (6).

Somewhat surprisingly, ballooning and fibrosis displayed a direct relationship with **α1-antitrypsin** in the model-based analysis (i.e. higher scores were associated with higher α1-antitrypsin levels), which was confirmed when reviewing the raw data. α1-antitrypsin levels in the population (31-296 mg/dL) were largely within or above the reference range. Merely 4.7% of the patients (28/600; 3.1% of total population) displayed values below 100 mg/dL (7), potentially due to genotypes causing α1-antitrypsin deficiency, which renders patients susceptible to liver disease (8). α1-antitrypsin deficiency may be masked by transient elevations of α1-antitrypsin in presence of inflammatory conditions or certain drugs (e.g. oral contraceptives) (9). Although a further report exists on higher α1-antitrypsin levels with higher liver damage, this finding requires further research (10).

In the current analysis, **hypertension** was associated with moderate to severe fibrosis (systolic blood pressure, portal hypertension) and portal chronic inflammation (portal hypertension). In a study by Mendes et al., 88% of NAFLD patients with portal hypertension displayed severe fibrosis or cirrhosis (11). For patients with absent/mild fibrosis, an association between portal hypertension and steatosis was additionally found, although the portal hypertension group was rather small (n=12 versus 192).

References

1. Loomba R, Abraham M, Unalp A, Wilson L, Lavine J, Doo E, et al. Association between diabetes, family history of diabetes, and risk of nonalcoholic steatohepatitis and fibrosis. Hepatology. 2012;56:943–51.

2. McPherson S, Hardy T, Henderson E, Burt AD, Day CP, Anstee QM. Evidence of NAFLD progression from steatosis to fibrosing-steatohepatitis using paired biopsies: Implications for prognosis and clinical management. J Hepatol. 2015;62:1148–55.

3. Wang Q, You H, Ou X, Zhao X, Sun Y, Wang M, et al. Non-obese histologically confirmed NASH patients with abnormal liver biochemistry have more advanced fibrosis. Hepatol Int. 2019;13:766–76.

4. Fabbrini E, Sullivan S, Klein S. Obesity and nonalcoholic fatty liver disease: biochemical, metabolic, and clinical implications. Hepatology. 2010;51:679–89.

5. Neuschwander-Tetri BA, Clark JM, Bass NM, Van Natta ML, Unalp-Arida A, Tonascia J, et al. Clinical, laboratory and histological associations in adults with nonalcoholic fatty liver disease. Hepatology. 2010;52:913–24.

6. Henson JB, Simon TG, Kaplan A, Osganian S, Masia R, Corey KE. Advanced fibrosis is associated with incident cardiovascular disease in patients with non-alcoholic fatty liver disease. Aliment Pharmacol Ther. 2020;51:728–36.

7. Ferrarotti I, Thun GA, Zorzetto M, Ottaviani S, Imboden M, Schindler C, et al. Serum levels and genotype distribution of α1-antitrypsin in the general population. Thorax. 2012;67:669–74.

8. Hamesch K, Mandorfer M, Pereira VM, Moeller LS, Pons M, Dolman GE, et al. Liver Fibrosis and Metabolic Alterations in Adults With alpha-1-antitrypsin Deficiency Caused by the Pi*ZZ Mutation. Gastroenterology. 2019;157:705-719.e18.

9. Janciauskiene SM, Bals R, Koczulla R, Vogelmeier C, Köhnlein T, Welte T. The discovery of α1-antitrypsin and its role in health and disease. Respir Med. 2011;105:1129–39.

10. Sirchak YS, Griga VI, Bedey NV, Pushkash II. Changes in the level of α1-antitrypsin in patients with non-alcoholic fatty liver disease. Wiad Lek. 2020;73:508–11.

11. Mendes FD, Suzuki A, Sanderson SO, Lindor KD, Angulo P. Prevalence and indicators of portal hypertension in patients with nonalcoholic fatty liver disease. Clin Gastroenterol Hepatol. 2012;10:1028-1033.e2.
